# ArcA overexpression induces fermentation and results in enhanced growth rates of ***E. coli***

**DOI:** 10.1038/s41598-017-12144-6

**Published:** 2017-09-19

**Authors:** Markus Basan, Sheng Hui, James R. Williamson

**Affiliations:** 1 Department of Systems Biology, Harvard Medical School, Boston, MA 02115 USA; 2 Department of Physics, University of California at San Diego, La Jolla, CA 92093 USA; 3 Department of Integrative Structural and Computational Biology, The Scripps Research Institute, La Jolla, CA 92037 USA; 4 The Skaggs Institute for Chemical Biology, The Scripps Research Institute, La Jolla, CA 92037 USA

## Abstract

Overflow metabolism in the presence of oxygen occurs at fast growth rates in a wide range of organisms including bacteria, yeast and cancer cells and plays an important role in biotechnology during production of proteins or metabolic compounds. As recently suggested, overflow metabolism can be understood in terms of proteome allocation, since fermentation has lower proteome cost for energy production than respiration. Here, we demonstrate that ArcA overexpression in aerobic conditions, results in downregulation of respiratory pathways and enhanced growth rates on glycolytic substrates of *E. coli*, coinciding with acetate excretion and increased carbon uptake rates. These results suggest that fermentation enables faster growth and demonstrate that fermentation on many glycolytic carbon sources is not limited by carbon uptake. Hence, these findings are difficult to reconcile with many alternative hypotheses that have been proposed for the origin of overflow metabolism and the growth rate dependence of fermentation and respiration, which are based on limited capacity of respiration or limitations in uptake rates and catabolic pathways. Instead, as suggested by increased lag phases of ArcA overexpression strains, respiratory energy metabolism may be related to a general preparatory response, observed for decreasing growth rates, but with limited advantages for maximizing steady-state growth rate.

## Introduction

Respiration and fermentation are alternative metabolic strategies for energy production. To supply energy required for their survival and growth, many organisms, including *E. coli*, use different combinations of respiration and fermentation depending on their environments and growth conditions^1–3^. Fermentation involves the excretion of byproducts such as acetate or lactate^4^, whereas using respiration, carbon is fully oxidized to CO_2_. As a consequence, a much lower amount of ATP per molecule of metabolized substrate is produced from fermentation compared to respiration. Nevertheless, fast-growing microbial cells, ranging from *E. coli* ^5^ and yeast^6^, and fast-growing mammalian cells like tumor cells or stem cells^7,8^, heavily rely on fermentation for their energy requirements, even in the presence of oxygen.

In previous work^3^, we demonstrated that the patterns of acetate excretion in *E. coli* under different growth limitations can be explained by a proteome allocation model. The metabolic advantage of fermentation over respiration is due to a lower investment in the protein machinery required to produce the necessary ATP flux for rapid growth. Hence, fermentation constitutes a “leaner” pathway, where abundant carbon can be “wasted” by excretion. Our previous study quantitatively explains interrelations between growth rates, carbon uptake rates, acetate excretion rates, as well as patterns of gene expression that are observed under diverse conditions. However, important questions regarding the underlying rationale of these patterns remain, in particular, the reason for the heavy reliance on respiration observed for many glycolytic carbon sources^3,9^, when fermentation should be advantageous for fast growth. It is unclear why *E. coli* strains grow slowly on many glycolytic substrates, and use respiration exclusively to meet their energy needs, even when the carbon source is highly abundant in the medium. Even at faster growth rates, it is unclear why bacteria use a combination of fermentation and respiration^3^, instead of concentrating all their resources on the “leaner” pathway. The proteome cost of most carbon specific operons is comparatively small^10^ and therefore, from the perspective of growth rate maximization, cells should be able to increase their uptake rates to take advantage of fermentation for energy production. Have *E. coli* strains simply not learned to express higher levels of carbon transporters on these substrates, or are there fundamental limitations to carbon uptake rates? Or do these slow growth rates arise from as yet unappreciated objectives other than growth rate maximization?

## Results

Here, to elucidate these questions, we directly tested the effect of repressing respiration on growth rates of *E. coli*. We demonstrate that forcing *E. coli* K12 strains to increase fermentation of glycolytic carbon sources results in enhanced growth rates, lower biomass yield and higher carbon uptake rates. Reflecting a lower protein cost fermentation, these results buttress our previous findings^3^, but challenge the commonly held notion that the heavy reliance on respiration for metabolism of many glycolytic carbon sources is caused by fundamental limitations in carbon availability or uptake.

A genetic construct was engineered that allowed repression of respiration in *E. coli*, by controlled overexpression of the global transcriptional regulator ArcA, using the linearly titrable P*tet*/*tetR* system^11^ (see Fig. S1). Normally, under anaerobic conditions, ArcA is phosphorylated by its membrane counterpart ArcB, and ArcA^P^ represses genes involved in respiration such as enzymes in the TCA cycle^12^. Here, we show that overexpression of ArcA results in downregulation of aerobic metabolic pathways even under aerobic conditions, forcing cells to rely more on fermentation for energy production.

**Figure 1:**
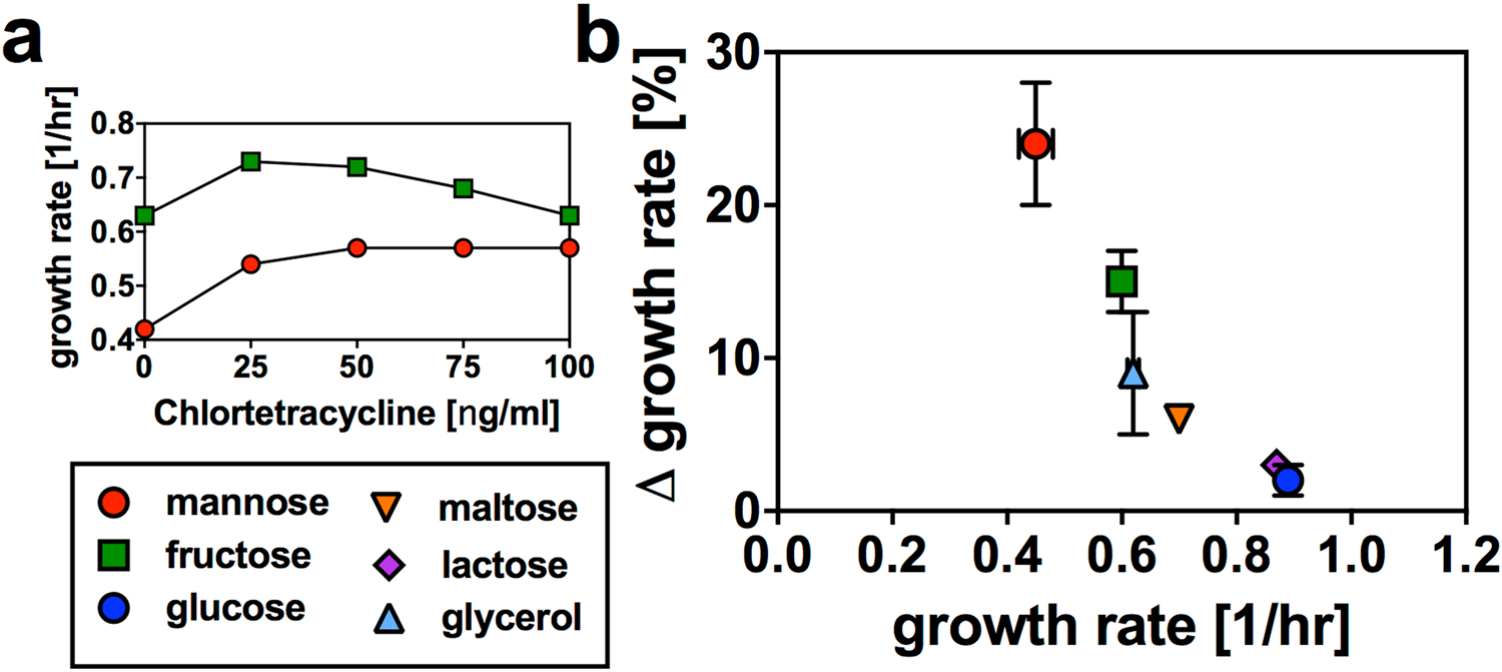
Growth rate improvement with ArcA overexpression. (**a**) Growth rates for different induction levels of ArcA overexpression on select glycolytic carbon sources. For many glycolytic carbon sources, we observed striking increases in growth (control strain, Fig. S1a, non-glycolytic carbon sources Fig. S2b). (**b**) Maximum relative growth rate improvement from titrated ArcA overexpression on different glycolytic carbon sources as a function of growth rate without ArcA overexpression. Data presented result from maxima observed in titration curves, as presented in panel a. The data is presented in Table S2. Error bars were established for a subset of carbon sources by measuring multiple biological repeats of calibration curves (mannose: 6 repeats; fructose: 6 repeats; glucose: 6 repeats; glycerol: 2 repeats). The data show growth rates on ‘slow’ glycolytic carbon sources could be significantly improved by ArcA overexpression.

Using this overexpression system, the growth physiology for different ArcA induction levels on minimal medium was characterized with various carbon sources, revealing a remarkable effect. While high ArcA expression levels were detrimental for growth, presumably by repressing the TCA cycle below levels required for the production of biomass precursors, intermediate ArcA levels resulted in significantly faster growth on many glycolytic carbon sources, as shown for fructose and mannose in Fig. 1a (control strain, Fig. S2a). Testing a range of glycolytic carbon sources with varying growth rates, improved growth rates were observed at intermediate ArcA overexpression levels for all carbons tested (Fig. 1b). Glycolytic carbons that allow fast growth rates of wildtype *E. coli* like glucose and lactose only exhibited a small, but reproducible improvement of their growth rates. But “slow” glycolytic carbon sources like mannose, on which wildtype *E. coli* exclusively utilizes respiratory pathways^1,10^, showed substantial growth rate improvements with intermediate ArcA overexpression. This improved growth rate of ArcA overexpression strains on glycolytic carbons was in marked contrast to the effect of ArcA for growth on TCA carbons such as succinate, where even moderate overexpression of ArcA had a strongly detrimental effect on growth rates (see Fig. S2b). The relative growth rate improvement as a function of the growth rate without ArcA overexpression is shown for different glycolytic carbon sources in Fig. 1b.

**Figure 2:**
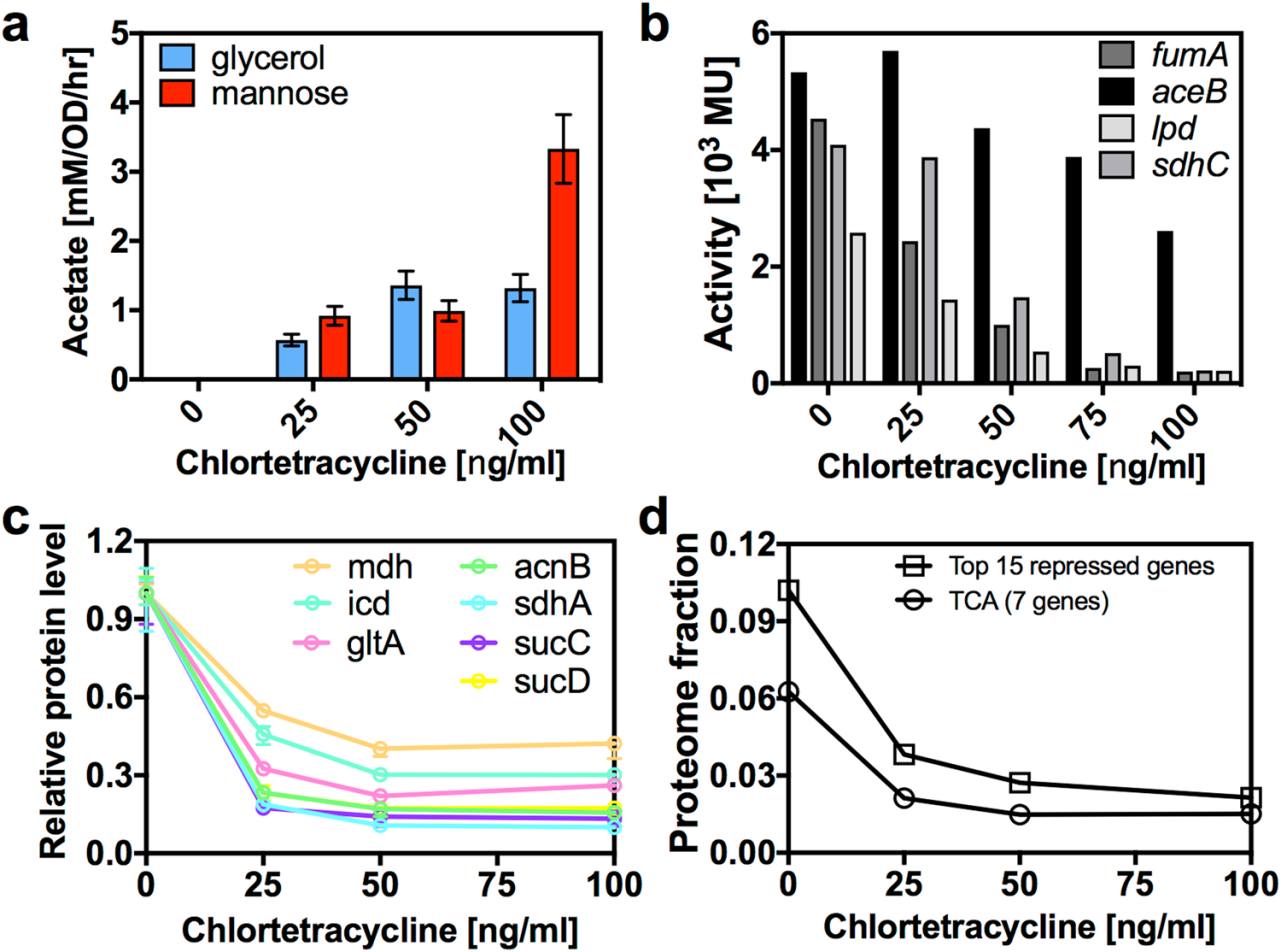
Effect of ArcA overexpression on acetate excretion and gene expression of respiratory pathways. (**a**) Acetate excretion rates on glycerol and mannose minimal medium with ArcA overexpression. Acetate excretion rates increase with increasing ArcA induction. Wildtype *E. coli* cells do not excrete acetate on either glucose or mannose^3^. (**b**) Effect of ArcA overexpression on gene expression of respiratory pathways for growth on fructose minimal medium measured via β-galactosidase reporters (Table S1). Respiratory genes are repressed by ArcA overexpression forcing the cell to rely on fermentation (panel a). (**c**) Proteomics results for ArcA overexpression on mannose minimal medium. Relative levels of TCA proteins with increasing ArcA induction. The level of ArcA is presented in Fig. S6. (**d**) Proteomics results for ArcA overexpression on mannose minimal medium. Estimates of the proteome fraction of the top 15 repressed proteins and also the 7 detected TCA proteins.

Improved growth rates suggest that ArcA overexpression results in the substitution of fermentation for respiration. To confirm this, the excretion rate of acetate, the main fermentation product of *E. coli*, was measured for different ArcA overexpression levels. Indeed, ArcA overexpression led to increased acetate excretion (Figs 2a and S3). In particular, acetate excretion appeared with poor carbon substrates such as mannose, where acetate production is absent in the wildtype^3^ and without ArcA induction.

The increase in growth rate and the excretion of fermentation products should coincide with a decrease in biomass yield and an increased carbon uptake rate. The saturating optical density (OD_600_) with low initial concentrations of different carbon sources was measured and for different levels of ArcA overexpression. As anticipated, cultures with ArcA overexpression ran out of carbon and stopped growing at lower culture densities (see Fig. S4). Together with the higher growth rates that we observe, this means that ArcA overexpression strains have higher carbon uptake rates. We note that acetate levels in these experiments are far below growth inhibitory concentrations.

To test if the growth rate enhancing effect of ArcA overexpression was specific to the NCM3722 strain^13^, the ArcA overexpression construct was introduced into the BW25113 background^14^. The enhanced growth rates and increased acetate excretion were also observed in this background (Fig. S5).

In order to directly study the effect of ArcA overexpression on gene expression, a set of β-galactosidase reporter constructs was engineered for genes involved in respiratory pathways. Expression levels measured this way are plotted against induction levels of ArcA overexpression in Fig. 2b. Indeed, ArcA overexpression was effective at repressing respiratory genes, affecting TCA genes more strongly than the glyoxylate shunt. Maximum growth rates were achieved for intermediate levels of respiratory genes. On the other hand, strong downregulation of respiratory genes often had a detrimental effect on growth rate, presumably because these pathways are required for the production of biomass precursors, as quantified in our previous work^3^. To gain a genome scale picture of the changes in gene expression resulting from ArcA overexpression, we employed quantitative proteomic mass spectrometry to track the relative changes in abundance of a large number of proteins (Fig. 2c). Consistent with the results from reporter constructs, proteomics revealed the global downregulation of respiratory pathways with ArcA overexpression. It is likely that the repression of non-essential proteins outside respiratory metabolism also contributes to the enhanced growth rates under ArcA overexpression (Fig. 2d).

Several other genetic alterations are known to lead to faster than wildtype growth rates on one or several carbons. These mutations include the knockout of flagellar genes (Δ*flhD*)^3^, growth on glycerol with mutations of an allosteric fructose 1,6-bisphosphate binding site in glycerol kinase GlpK^15^, as well as recent findings on the optimal level of cAMP^16^. The knockout of the global transcriptional regulator of glycolysis and gluconeogenesis *cra* exhibits similarly striking improvements in growth rate for a number of glycolytic carbon sources. Δ*cra* strains cannot grow on many gluconeogenic carbon sources, as transcriptional activity of cra represses glycolytic enzymes and activates gluconeogenesis^17,18^. Therefore, we suspected that the high growth rate, fermentative phenotype of ArcA overexpression could be enhanced by the cra knockout. The ArcA overexpression system was introduced into a Δ*cra*-background, and growth on mannose minimal medium was tested. Indeed, the effects of Δ*cra* and ArcA overexpression were additive, with a resulting growth rate that was roughly twice as fast as the growth rate of the wildtype strain on mannose minimal medium (Fig. S7).

Our results demonstrate that downregulation of respiratory pathways via controlled overexpression of the global regulator ArcA in *E. coli*, remarkably leads to faster growth rates on a large number of glycolytic carbon sources. The largest increase in growth rate was observed for ‘slow’ glycolytic carbon sources such as mannose, on which wildtype *E. coli* normally exclusively relies on respiration for energy production. Acetate excretion could be induced even on these ‘slow’ carbon sources and hence, the threshold growth rate for acetate excretion was decreased by ArcA overexpression^3^. The increase in growth rates with ArcA overexpression coincided with increased acetate excretion, a decreased biomass yield and increased carbon uptake rates. The downregulation of genes involved in respiration by ArcA overexpression was verified using reporters of gene expression, as well as proteomics.

## Discussion

We note that it may be possible to increase growth rates even more, because ArcA overexpression likely does not result in perfect substitution of respiration by fermentation, including sufficiently high uptake rates, required for perfect compensation of the loss of respiraton. ArcA overexpression may also have regulatory effects beyond energy metabolism. However, our results demonstrate that the upregulation of respiration pathways on “slow” glycolytic carbons is not caused by limitations of carbon uptake, as commonly believed, because significantly faster growth rates fueled by even higher uptake rates are evidently possible. Instead, we argue that the observed utilization of respiratory pathways like the TCA and glyoxylate cycle on slow glycolytic carbon sources^1^, as well as other recent findings regarding suboptimal growth rates^16^, arise from different objectives﻿, other than maximizing steady-state growth rate, and may be part of a global preparatory response for adaptation to changing environments. As one example, in a study in preparation, we show a direct dependence of lag times resulting from carbon shifts on growth rates and glycolytic flux, with fast growth rates resulting in very long lag phases, consistent with previous findings^19^ and with growth rate improvements of the cra knockout (Fig. S7). Indeed, ArcA overexpression before carbon shifts resulted in substantially increased lag times (Fig. S8).

Finally, we note that ArcA overexpression or ArcB activation^12,20^, which shows even stronger repression of respiration, should be a useful tool for biotechnology applications, like for the production of metabolites. At high levels, ArcA/ArcB activity fully represses unwanted biomass production and virtually stops growth, while resulting in a large fermentative metabolic flux that could be diverted to desired products. This is similar to the effect of anaerobic conditions, a common strategy for maximizing production yield, but ArcAB activation can result in a stronger inhibition of biomass production and has the added advantage of allowing efficient energy metabolism via the electron transport chain.

## Methods

### Strain construction

#### Construction of ArcA overexpression strains

The *arcA* gene including the 5’-UTR was amplified from genomic DNA of NCM3722 using primers Ptet-ArcA-XhoI-F and ArcA-BamHI-R. The P*tet* promoter sequence, as well as an *XhoI* site were included in the forward primer Ptet-ArcA-XhoI-F. A *BamHI* site was included in the reverse primer ArcA-BamHI-R. The resulting DNA fragment was used to replace P*tet*-*gfp* in the plasmid pZA31-*gfp*(Levine *et al*., 2007) yielding the plasmid pZA31 Ptet-*arcA*. This plasmid was then transformed to various backgrounds. The plasmid pZA31 P*tet*-*RF* was characterized in a previous study(Levine *et al*., 2007).

#### Chromosomal PaceB-lacZ, PfumA-lacZ, PsdhC-lacZ, Plpd-lacZ fusions

The *aceB* promoter region (−191 bp to +204 bp relative to the *aceB* translational start point), the *fumA* promoter region (−426 bp to +216 bp relative to the *fumA* translational start point), the *sdhC* promoter region (−231 bp to +380 bp relative to the *sdhC* translational start point) and the *lpd* promoter region (−222 bp to +359 bp relative to the *lpd* translational start point) were amplified from genomic DNA of NCM3722. These DNA fragments were then inserted between the *Xho*I and BamHI sites downstream of the rrnB terminator (*rrnB*T) in pKDT(Klumpp *et al*., 2009), yielding the plasmids pKDT-P*aceB*, pKDT-P*fumA* and pKDTP*sdhC*, respectively. Using each of these plasmids as template, the DNA region containing the *km* ^*r*^ gene, *rrnB*T and the promoter was PCR amplified and subsequently integrated into the chromosome of *E. coli* strain NQ309 to replace part of *lacI* and the entire P*lacZ* (from +134 bp after the *lacI* translational start codon to the *lacZ* translational start codon)(Datsenko & Wanner, 2000). Then each of the promoter-*lacZ* fusions was transferred to different genetic backgrounds by phage P1 *vir* mediated transduction.

#### Construction of the cra deletion strain (NQ1077)

The Δ*cra* deletion allele in strain LJ2801 (*E. coli* Genetic Stock Center, Yale Univ.), in which a *km* ^*r*^ gene is substituted for the *cra* gene, was transferred to wildtype NCM3722, resulting in the strain NQ1077.

### Growth of bacterial culture

#### Growth media

NC- minimal medium (Csonka *et al*., 1994), which contains K2SO4 (1 g), K2HPO4.3H2O (17.7 g), KH2PO4 (4.7 g), MgSO4.7H2O (0.1 g), and NaCl (2.5 g) in one liter, and is supplemented with various carbon and nitrogen sources. For carbon limited growth of wild-type cells, 20 mM NH4Cl were used. For carbon sources concentrations were based on the number of carbon atoms in the molecule: 20 mM for C_6_-carbons, 30 mM for C_4_-carbons and 40 mM for C_3_-carbons.

#### Growth measurements

Batch culture growth was performed in a 37 °C water bath shaker shaking at 250 rpm. The culture volume was at most 10 ml in 25 mm × 150 mm test tubes. Each growth experiment was carried out in three steps: seed culture in LB broth, pre-culture and experimental culture in identical minimal medium. For seed culture, one colony from fresh LB agar plate was inoculated into liquid LB and cultured at 37 °C with shaking. Cells were then diluted into the minimal medium and cultured in 37 °C water bath shaker overnight (pre-culture). The overnight pre-culture was allowed to grow for at least 3 doublings. Cells from the overnight pre-culture was then diluted to OD_600_ = 0.005–0.025 in identical pre-warmed minimal medium, and cultured in 37 °C water bath shaker (experimental culture). 200 μl cell culture was collected in a Sterna Sub-Micro Cuvette for OD_600_ measurement using a Thermal Spectrophotometer every half doubling of growth. The time taken for each sample collection is <30 sec and had no measureable effect on cell growth.

#### β-Galactosidase Assay

The assay was performed following a similar protocol as detailed in a previous study^6^. 100*μ*L samples were taken for at least 4 different times during exponential growth (typically at OD_600_ between 0.1 and 0.6). LacZ samples were immediately added to 0.9 mL of freshly prepared Z-buffer (in 1 L: 8.52 g Na_2_HPO_4_, 5.5 g NaH_2_PO_4•_H_2_O, 0.75 g KCl and 0.25 g MgSO_4•_7H_2_O, with 0.004%(w/v) SDS and 40 mM *β*-mercaptoethanol) with 100 *μ*L chlorophorm. Cells were disrupted by vortexing and stored at 4 °C until all samples were collected. After all samples were collected, they were briefly vortexed a second time. After 5–10 minutes at room temperature to settle the chlorophorm, the lysates were diluted (typically 1:4) in Z-buffer. 200 *μ*L was then added to a 96-well plate (Sarstedt). Immediately prior to reading in GENiosPro (Tecan) plate reader, 40 *μ*L of of 4 mg/mL Ortho-Nitrophenyl-*β*-galactoside (Sigma) in 0.1 M phosphate buffer (pH = 7.0) was added to each well. The plate reader was set to read absorbance at a wavelength of 420 nm every minute for 60 to 120 minutes at 28 °C.

The slope of the plot of OD_420_ vs. time (in minutes) for all replicates was used to calculate the *β*-galactosidase activity (Units/mL) in the original sample via the following conversion:

$$\beta \mbox{-}{\rm{galactosidase}}\,{\rm{activity}}\,({\rm{Units}}/{\rm{mL}})=1000\times \frac{O{D}_{420}}{\min }\times ({\rm{fold}}\,{\rm{dilution}})\times 2.66,$$

where the fold dilution is from both the initial dilution of cell sample in Z-buffer/chloroform and the dilution of lysates in Z-buffer before being loaded to the plate. The factor 2.66 converts the plate-reader data to the activity obtained using the original assay protocol by Miller, and is specific to the path-length through the sample (*i.e*. 240 *μ*L in a 96-well plate). The slope of the plot of activity (Units/mL) versus the sample OD_600_ yields theactivity in Miller units (Units/mL/OD_600_).

### Acetate assay

#### Acetate assay

200 *μ*L samples were taken and immediately frozen. Before the assay, samples were thawed in water and immediately centrifuged at maximum speed (15,000 rpm) for 2.5 min. 100 *μ*L of supernatant were used to measure acetate concentrations using the Acetate Assay kit (Catalog #:10148261035, R-Biopharm).

### Proteomic mass spectrometry

#### Sample collection

For each of the cultures, 1.8 ml of cell culture at OD_600_ = 0.4~0.5 was collected by centrifugation. The cell pellet was re-suspended in 0.2 ml water and fast frozen on dry ice.

#### Sample preparation

Aliquot of the mixture of the two ^15^N labeled cell samples was mixed with each of the non-labeled cell samples, which contained the same amount of proteins (~100 µg proteins). Proteins were precipitated by adding 100% (w/v) trichloroacetic acid (TCA) to 25% final concentration. Samples were let stand on ice for a minimum of 1 hour. The protein precipitates were sped down by centifugation at 16,000 g for 10 min at 4 °C. The supernatant was removed and the pellets were washed with cold acetone. The pellets were dried in a Speed-Vac concentrator. The pellets were dissolved in 80 µl 100 mM NH_4_HCO_3_ with 5% acetonitrile (ACN). Then 8 µl of 50 mM dithiothreitol (DTT) was added to reduce the disulfide bonds before the samples were incubated at 65 °C for 10 min. Cysteine residues were modified by the addition of 8 µl of 100 mM iodoacetamide (IAA) followed by incubation at 30 °C for 30 min in the dark. The proteolytic digestion was carried out by the addition of 8 µl of 0.1 µg/µl trypsin (Sigma-Aldrich, St. Louis, MO) with incubation overnight at 37 °C. The peptide solutions were cleaned by using the PepClean® C-18 spin columns (Pierce, Rockford, IL). After drying in a Speed-Vac concentrator, the peptides were dissolved into 10 µL sample buffer (5% ACN and 0.1% formic acid).

### Proteomics

#### Mass spectrometry

The peptide samples were analyzed on an Agilent G6520B TOF mass spectrometer. Peptides were eluted from a 90-min, 5–60% concave acetonitrile gradient. The precursor mass window was from 400 to 2000 m/z and the product ion mass window from 80 to 2000 m/z. Fragmentation were collected under the data-dependent Auto MS/MS mode with 1 sec scan cycle times, 4 maximum precursors per cycle, a 1500 abundance threshold, and active exclusion after 2 spectra for 5 min.

#### Protein identification

The raw mass spectrometry data files generated by the Agilent G6520B TOF system were converted to Mascot generic format (mgf) files, which were submitted to the Mascot database searching engine (Matrix Sciences, London, UK) against the *E. coli* SwissProt database to identify proteins. The following parameters were used in the Mascot searches: maximum of two missed trypsin cleavage, fixed carbamidomethyl modification, variable oxidation modification, peptide tolerance ±0.1 Da, MS/MS tolerance ±0.1 Da, and 1+, 2+, and 3+ peptide charge. All peptides with scores less than the identity threshold (P = 0.05) were discarded.

#### Relative protein quantitation

The raw mass spectrometry data files were converted to the.mzMLfiles. Results of the Mascot search were submitted with.mzML files to our in-house quantification software(Sperling *et al*., 2008). Briefly, intensity is collected for each peptide over a box in RT and m/z space that encloses the envelope for the light and heavy peaks. The data is collapsed in the RT dimension and the light and heavy peaks are fit to a multinomial distribution (a function of the chemical formula of each peptide) using a least squares Fourier transform convolution routine(Sperling *et al*., 2008), which yields the relative intensity of the light and heavy species. The ratio of the non-labeled to labeled peaks was obtained for each peptide in each sample.

The relative protein quantitation data for each protein in each sample mixture was then obtained as a ratio by taking the median of the ratios of its peptides. No ratio (i.e., no data) was obtained if there was only one peptide for the protein. The uncertainty for each ratio was defined as the two quartiles associated with the median. To filter out data with poor quality, the ratio was removed for the protein in that sample if at least one of its quartiles lied outside of 50% range of its median; Furthermore, ratios were removed for a protein in all the sample mixtures in a growth limitation if at least one of the ratios has one of its quartiles lying outside of the 100% range of the median.

#### Proteome fraction estimation

Proteome fractions of cells grown on mannose minimal medium with no chlortetracycline were taken from previously quantified proteome fractions for carbon-limiting cells that have the same growth rate (NCM3722-background strain with glycerol uptake titrated)(Hui *et al*., 2015). The proteome fraction for a protein in cells with a given level of chlortetracycline was then calculated by multiplying the protein’s proteome fraction in the zero-chlortetracycline condition with its expression level relative to the zero-chlortetracycline condition.

### Error analysis

To establish our central finding (error bars in Fig. 1b, main text), the increased growth rate with ArcA overexpression, growth rate curves versus inducer curves for ArcA overexpression were repeated independently multiple times for multiple carbon sources (mannose: 6 repeats; fructose: 6 repeats; glucose: 6 repeats; glycerol: 2 repeats). Error bars for saturating OD_600_ were established from repeats on mannose minimal medium (Fig. S4). Based on experience from previous work (Basan *et al*., 2015), for measurements of acetate excretion rates resulting from four acetate measurements along the growth curve, we assumed 15% relative errors.

## Electronic supplementary material

Supplementary Information

## References

[1] Gerosa, L., Rijsewijk, B. R. B. H. Van & Christodoulou, D. Pseudo -transition analysis identifies active transcriptional and metabolic regulators that govern bacterial nutrient adaptations. 1–34

[2] Schuetz R, Zamboni N, Zampieri M, Heinemann M, Sauer U (2012). Multidimensional optimality of microbial metabolism. Science.

[3] Basan M (2015). Overflow metabolism in Escherichia coli results from efficient proteome allocation. Nature.

[4] Neidhardt, F. C., 1931-, Ingraham, J. L. & Schaechter, M. Physiology of the bacterial cell. (1990).

[5] Wolfe AJ (2005). The acetate switch. Microbiol. Mol. Biol. Rev..

[6] De Deken RH (1966). The Crabtree Effect: A Regulatory System in Yeast. J. Gen. Microbiol..

[7] Vander Heiden MG, Cantley LC, Thompson CB (2009). Understanding the Warburg effect: the metabolic requirements of cell proliferation. Science.

[8] Hanahan D, Weinberg RA (2011). Hallmarks of cancer: The next generation. Cell.

[9] Gerosa L (2015). Pseudo-transition Analysis Identifies the Key Regulators of Dynamic Metabolic Adaptations from Steady-State Data. Cell Syst..

[10] Hui, S. *et al*. Quantitative proteomic analysis reveals a simple strategy of global resource allocation in bacteria. *Mol. Syst. Biol*. (2015). 10.15252/msb.20145697 PMC4358657 25678603

[11] Klumpp S, Zhang Z, Hwa T (2009). Growth rate-dependent global effects on gene expression in bacteria. Cell.

[12] Alvarez AF, Georgellis D (2010). In vitro and *in vivo* analysis of the ArcB/A redox signaling pathway. Methods Enzymol..

[13] Brown, S. D. & Jun, S. Complete Genome Sequence of Escherichia coli NCM3722. *Genome Announc*. **3** (2015). 10.1128/genomeA.00879-15 PMC4541272 26251500

[14] Grenier, F., Matteau, D., Baby, V. & Rodrigue, S. Complete Genome Sequence of Escherichia coli BW25113. *Genome Announc*. **2** (2014). 10.1128/genomeA.01038-14 PMC4200154 25323716

[15] Applebee MK, Joyce AR, Conrad TM, Pettigrew DW, Palsson BØ (2011). Functional and metabolic effects of adaptive glycerol kinase (GLPK) mutants in Escherichia coli. J. Biol. Chem..

[16] Towbin BD (2017). Optimality and sub-optimality in a bacterial growth law. Nat. Commun..

[17] Cortay JC (1994). *In vitro* asymmetric binding of the pleiotropic regulatory protein, FruR, to the ace operator controlling glyoxylate shunt enzyme synthesis. J. Biol. Chem..

[18] Ramseier TM (1996). Cra and the control of carbon flux via metabolic pathways. Res. Microbiol..

[19] Kotte O, Volkmer B, Radzikowski JL, Heinemann M (2014). Phenotypic bistability in Escherichia coli’s central carbon metabolism. Mol. Syst. Biol..

[20] Malpica R, Franco B, Rodriguez C, Kwon O, Georgellis D (2004). Identification of a quinone-sensitive redox switch in the ArcB sensor kinase. Proc. Natl. Acad. Sci. USA..

