# Supplementary material for: ArcA overexpression induces fermentation and results in enhanced growth rates of E. coli
Source: Sci Rep. 2017 Sep 19;7:11866. doi: 10.1038/s41598-017-12144-6 (PMC5605494; doi:10.1038/s41598-017-12144-6)
Supplement: Supplementary file 1 — Supplementary Information [file 41598_2017_12144_MOESM1_ESM.pdf]

ArcA overexpression induces fermentation and results in enhanced growth rates of *E. coli*

Markus Basan, Sheng Hui and James R. Williamson

Supplementary Information

Strain	Genotype	Description
NCM3722	wild-type *E. coli* K12 strain	parent strain [1]
BW25113	wild-type *E. coli* K12 strain	parent strain [2]
NQ1280	NCM3722, attB::Spc-P*tet-tetR-lacIq*, pZA31 P*tet-arcA*	ArcA overexpression in NCM3722
NQ1546	NCM3722, attB::Spc-P*tet-tetR-lacIq*, pZA31 P*tet*-RF	Control strain, overexpression of a random protein fragment [3].
NQ1281	NCM3722, attB::Spc-P*tet-tetR-lacIq*, pZA31 P*tet-arcA*, Δ*cra-km*^{R}	ArcA overexpression & Δ*cra*
NQ1547	BW25113, attB::Spc-P*tet-tetR-lacIq*, pZA31 P*tet-arcA*	ArcA overexpression in BW25113
NQ1548	NCM3722, attB::Spc-P*tet-tetR-lacIq*, pZA31 P*tet-arcA*, *km*^{R}-P*lacZ*::P*sdhC*	ArcA overexpression, P*sdhC* reporter
NQ1549	NCM3722, attB::Spc-P*tet-tetR-lacIq*, pZA31 P*tet-arcA*, *km*^{R}-P*lacZ*::P*fumA*	ArcA overexpression, P*fumA* reporter
NQ1550	NCM3722, attB::Spc-P*tet-tetR-lacIq*, pZA31 P*tet-arcA*, *km*^{R}-P*lacZ*::P*lpd*	ArcA overexpression, P*lpd* reporter
NQ1551	NCM3722, attB::Spc-P*tet-tetR-lacIq*, pZA31 P*tet-arcA*, *km*^{R}-P*lacZ*::P*aceB*	ArcA overexpression, P*aceB* reporter
NQ1077	NCM3722, Δ*cra-km*^{R}	*cra* knockout
NQ1225	NCM3722, Δ*flhD-km*^{R}	*flhD* knockout

Table S1: Strains used in this study. *E. coli* K-12 strain NCM3722[4,5] provided kindly by Sydney Kustu lab. See Supplementary Methods for details of strain construction.

Strain	Medium	No induction growth rate (1/hr)		Maximum growth rate (1/hr)		Growth rate improvement (%)	
		**μ**	**σ**	**μ**	**σ**	**μ**	**σ**
NCM3722	Mannose	0.45	0.03	0.55	0.03	24	4
	fructose	0.60	0.02	0.69	0.03	15	2
	glucose	0.89	0.01	0.91	0.01	2	1
	maltose	0.70	-	0.74	-	6	-
	lactose	0.87	-	0.90	-	3	-
	glycerol	0.62	0.01	0.68	0.03	9	4

Table S2: Data underlying Fig. 1b. Data presented resulted from titration curves, as presented in Fig. 1a. Number of biological repeats of calibration curves were as follows: mannose: 6; fructose: 6; glucose: 6; maltose: 1; lactose: 1; glycerol: 2.

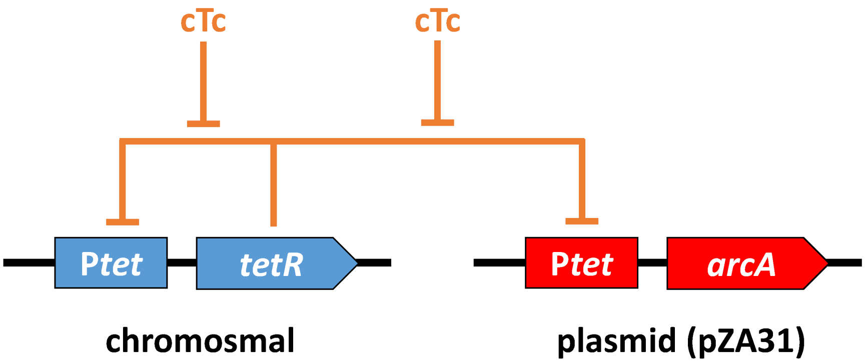

Fig. S1: ArcA overexpression system. The overexpression system consists of the pZA31 plasmid[3] carrying the *arcA* gene driven by the P*tet* promoter and a chromosomal P*tet* promoter driving TetR expression. The system can be induced via the addition of chlortetracycline, which releases autorepression by TetR. The P*tet*/TetR system constitutes a tight and linearly inducible system, as characterized previously[6].

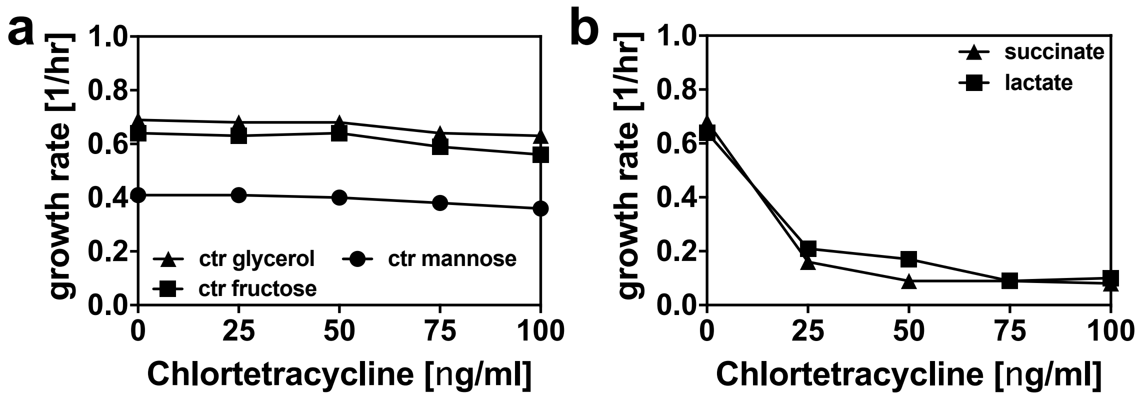

Fig. S2: Control construct and non-glycolytic carbons. a, We overexpress a random protein fragment instead of ArcA from an otherwise identical expression system (Fig. S1) as a control. The control construct does not exhibit the growth enhancing effect of ArcA overexpression. **b,** ArcA overexpression on non-glycolytic carbon sources. In contrast to glycolytic carbons, ArcA overexpression is highly detrimental for growth on these carbon sources.

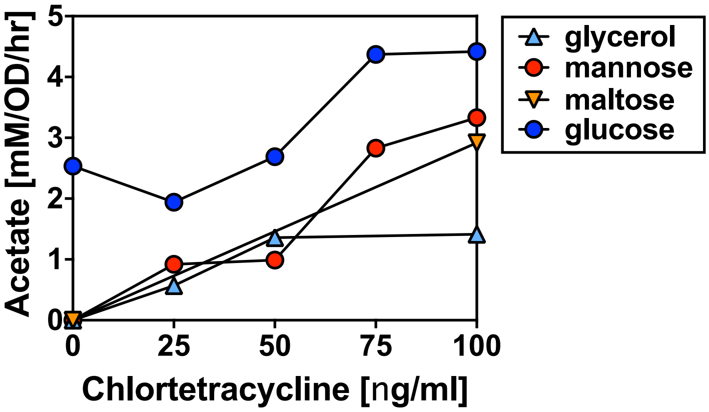

Fig. S3: Acetate excretion with ArcA overexpression. Acetate excretion rates with different levels of ArcA induction and different carbon sources. ArcA overexpression induces higher fermentation for glycolytic carbon sources.

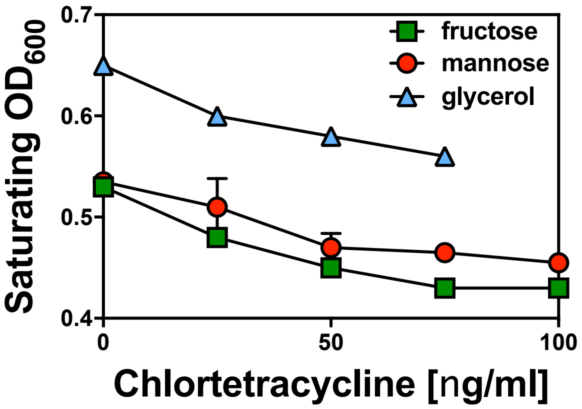

Fig. S4: Saturating OD_{600} with ArcA overexpression. Saturating optical density in the culture (OD_{600}) with low carbon concentrations (0.05% w/v) as a function of ArcA induction level. ArcA overexpression results in faster growth rates (Fig. 1) in combination with acetate excretion (Fig. 2a & Fig. S3). This result also demonstrates that carbon uptake rate increases with ArcA overexpression, because inoculated from the same initial OD_{600} total carbon runs out at a lower culture density OD_{sat} and the culture reaches OD_{sat} more quickly (higher growth rate) with intermediate ArcA overexpression (uptake rate is roughly given by growth rate*total carbon/OD_{sat}). Error bars from repeats are included for all induction levels on mannose.

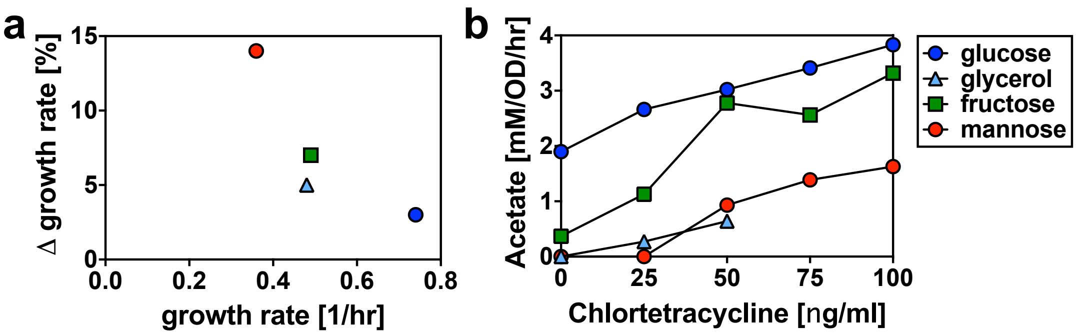

Fig. S5: Effect of ArcA overexpression in BW25113 strain. We performed controlled ArcA overexpression in a BW25113 background to exclude strain specific effects. **a,** Maximum relative growth rate improvement in the BW25113 strain from titrated ArcA overexpression on different glycolytic carbon sources as a function of growth rate without ArcA overexpression. Growth rates on 'slow' glycolytic carbon sources could be significantly improved by ArcA overexpression, similar to the effect of ArcA overexpression in NCM3722 (Fig. 1b, main text). **b,** Acetate excretion rates of BW25113 on glucose, glycerol, fructose and mannose minimal medium with ArcA overexpression. Acetate excretion rate increases with increasing ArcA induction similar to the result in NCM3722 (Fig. 2a, main text).

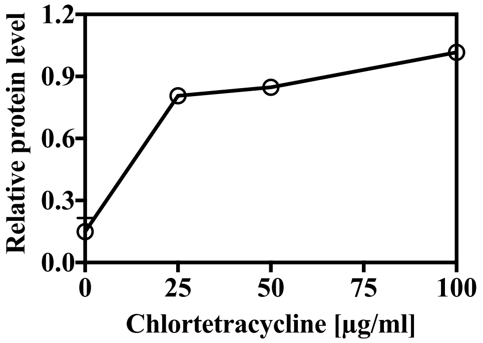

Fig. S6: ArcA protein level measured via proteomics on mannose. Relative levels of ArcA on mannose minimal medium versus inducer concentration.

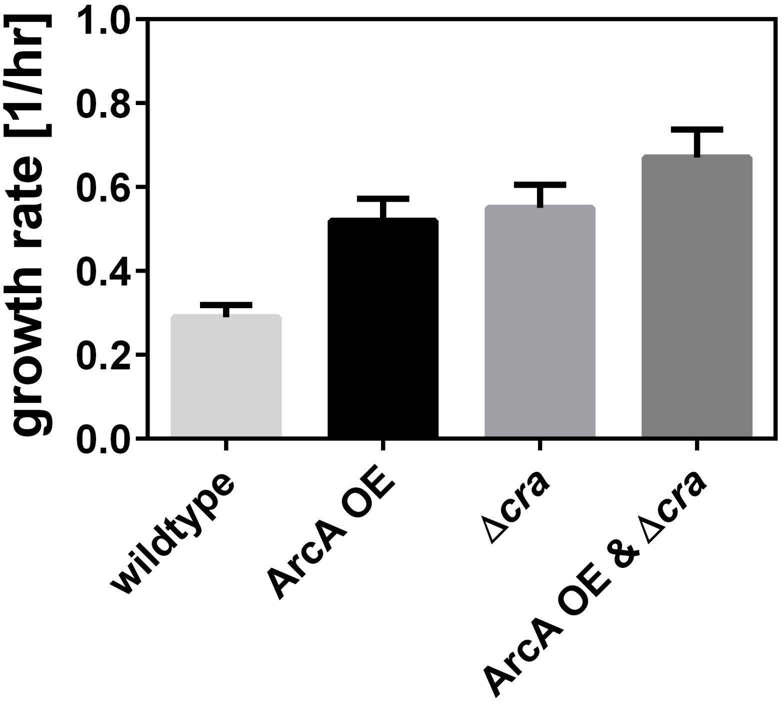

Fig. S7: Growth rate improvements for *cra* knockout and ArcA overexpression. Growth rates on mannose minimal medium for wildtype NCM3722, ArcA overexpression, *cra* knockout and the combination of ArcA overexpression and *cra* knockout. The growth rate improvements resulting from ArcA overexpression and *cra* knockout are comparable. Their effects are additive, as the combination of ArcA overexpression and *cra* knockout results in even faster growth. We note that the slower growth rates in these experiments results from the fact that they were carried out in mannose minimal medium in a higher range of culture OD_{600}, where mannose exhibits a slower characteristic growth rate.

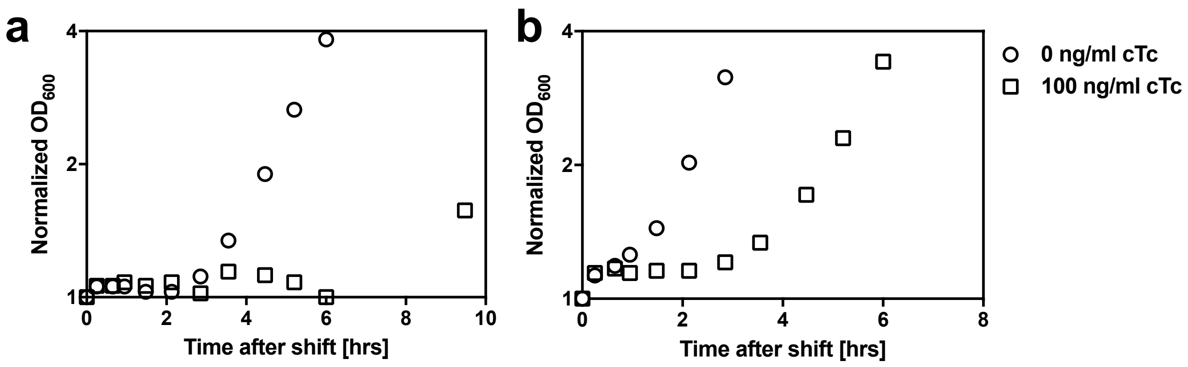

Fig. S8: Growth curves resulting from ArcA overexpression before carbon shifts. ArcA overexpression in preshift conditions results in significantly increased lag times. a, Shift from glucose to acetate minimal medium. b, Shift from glucose to succinate minimal medium. Bacteria were grown in batch culture with no induction induction of ArcA overexpression (circles) and alternatively strong induction (squares) (effect on growth rate before the shift were negligible). Cell were harvested by centrifugation and washed once with warm postshift medium before being gently resuspended in warm postshift medium.

Suppl. References

1. Brown, S. D. & Jun, S. Complete Genome Sequence of Escherichia coli NCM3722. *Genome Announc.* **3,** (2015).
2. Grenier, F., Matteau, D., Baby, V. & Rodrigue, S. Complete Genome Sequence of Escherichia coli BW25113. *Genome Announc.* **2,** (2014).
3. Levine, E., Zhang, Z., Kuhlman, T. & Hwa, T. Quantitative characteristics of gene regulation by small RNA. *PLoS Biol.* **5,** e229 (2007).
4. Soupene, E. *et al.* Physiological studies of Escherichia coli strain MG1655: growth defects and apparent cross-regulation of gene expression. *J. Bacteriol.* **185,** 5611–26 (2003).
5. Lyons, E., Freeling, M., Kustu, S. & Inwood, W. Using genomic sequencing for classical genetics in E. coli K12. *PLoS One* **6,** e16717 (2011).
6. Klumpp, S., Zhang, Z. & Hwa, T. Growth rate-dependent global effects on gene expression in bacteria. *Cell* **139,** 1366–75 (2009).
